# The mixed-metal tris­(di­sulfide) thio­phosphate, KNb_1.77_Ta_0.23_PS_10_

**DOI:** 10.1107/S1600536814000592

**Published:** 2014-01-15

**Authors:** Yoonjeong Lee, Woojin Yoon, Hoseop Yun

**Affiliations:** a Division of Energy Systems Research and Department of Chemistry, Ajou University, Suwon 443-749, Republic of Korea

## Abstract

The title compound *catena*-poly[potassium [tri-μ-disulfido-μ-tetra­thiophos­pha­to-di[niobate(IV)/tantalate(IV)(0.885/0.115)]]], has been obtained through the reaction of the elements with KCl. The title compound is isostructural with KNb_2_PS_10_, with the Nb sites occupied by statistically disordered Nb (88.5%) and Ta (11.5%) atoms. The structure is composed of anionic _∞_^1^[*M*_2_PS_10_]^−^ chains along [100] (*M* = Nb/Ta) and K^+^ ions. This chain is built up from distorted bicapped trigonal prisms [*M*S_8_] and [PS_4_] tetra­hedra. There are no inter­chain bonding inter­actions, except for electrostatic and van der Waals forces. The S_2_^2−^ and S^2−^ anionic species and the *M* ^4+^–*M* ^4+^ pair [*M*—*M* = 2.8939 (3) Å] are observed. The classical charge balance is represented by [K^+^][*M* ^4+^]_2_[PS_4_^3−^][S_2_^2−^]_3_.

## Related literature   

For the related mixed-metallic phase KNb_1.75_V_0.25_PS_10_, see: Yu & Yun (2011 ▶). For related quaternary compounds, see: Goh *et al.* (2002 ▶); Do & Yun (1996 ▶, 2009 ▶); Kim & Yun (2002 ▶); Kwak *et al.* (2007 ▶); Bang *et al.* (2008 ▶) and for quintenary compounds, see: Kwak & Yun (2008 ▶); Dong *et al.* (2005*a* ▶,*b* ▶). For Cs_0.5_Ag_0.5_Nb_2_PS_10_, see: Park & Yun (2010 ▶). For a typical Nb^4+^—Nb^4+^ bond length, see: Angenault *et al.* (2000 ▶).

## Experimental   

### 

#### Crystal data  

KNb_1.77_Ta_0.23_PS_10_
*M* *_r_* = 596.52
Orthorhombic, 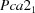

*a* = 13.0049 (3) Å
*b* = 7.5262 (2) Å
*c* = 13.3616 (3) Å
*V* = 1307.81 (6) Å^3^
*Z* = 4
Mo *K*α radiation
μ = 5.45 mm^−1^
*T* = 290 K
0.32 × 0.06 × 0.04 mm

#### Data collection  

Rigaku R-AXIS RAPID S diffractometer
Absorption correction: multi-scan (*ABSCOR*; Higashi, 1995 ▶) *T*_min_ = 0.724, *T*_max_ = 1.000
12074 measured reflections
2974 independent reflections
2842 reflections with *I* > 2σ(*I*)
*R*_int_ = 0.031

#### Refinement  

*R*[*F* ^2^ > 2σ(*F* ^2^)] = 0.019
*wR*(*F* ^2^) = 0.040
*S* = 1.08
2974 reflections
130 parameters
1 restraint
Δρ_max_ = 0.40 e Å^−3^
Δρ_min_ = −0.64 e Å^−3^
Absolute structure: Flack (1983 ▶)
Absolute structure parameter: 0.102 (14)

### 

Data collection: *RAPID-AUTO* (Rigaku, 2006 ▶); cell refinement: *RAPID-AUTO*; data reduction: *RAPID-AUTO*; program(s) used to solve structure: *SHELXS97* (Sheldrick, 2008 ▶); program(s) used to refine structure: *SHELXL97* (Sheldrick, 2008 ▶); molecular graphics: locally modified version of *ORTEP* (Johnson, 1965 ▶); software used to prepare material for publication: *WinGX* (Farrugia, 2012 ▶).

## Supplementary Material

Crystal structure: contains datablock(s) I, New_Global_Publ_Block. DOI: 10.1107/S1600536814000592/ru2056sup1.cif

Structure factors: contains datablock(s) I. DOI: 10.1107/S1600536814000592/ru2056Isup2.hkl

CCDC reference:

Additional supporting information:  crystallographic information; 3D view; checkCIF report

## Figures and Tables

**Table 1:** Selected bond lengths (Å)

Nb1/Ta1—S8^i^	2.4753 (10)
Nb1/Ta1—S7^ii^	2.4895 (9)
Nb1/Ta1—S2^iii^	2.5133 (10)
Nb1/Ta1—S9^i^	2.5255 (9)
Nb1/Ta1—S6^i^	2.5545 (9)
Nb1/Ta1—S10^ii^	2.5653 (8)
Nb1/Ta1—S1	2.5831 (9)
Nb1/Ta1—S4	2.6438 (8)
Nb2/Ta2—S9^iv^	2.4742 (9)
Nb2/Ta2—S2^i^	2.4805 (9)
Nb2/Ta2—S8^iv^	2.5185 (10)
Nb2/Ta2—S7	2.5513 (9)
Nb2/Ta2—S6^i^	2.5568 (9)
Nb2/Ta2—S10^ii^	2.5597 (8)
Nb2/Ta2—S5	2.5802 (8)
Nb2/Ta2—S4	2.6369 (8)
P—S3	1.9725 (14)
P—S5	2.0470 (14)
P—S1	2.0568 (13)
P—S4	2.0888 (14)
S2—S8^ii^	2.0302 (16)
S6—S10^v^	2.0568 (13)
S7—S9^iv^	2.0452 (15)

Symmetry codes: (i) 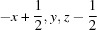; (ii) 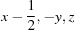; (iii) 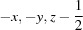; (iv) 
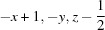
; (v) 
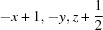
.

**Fig. 1.**
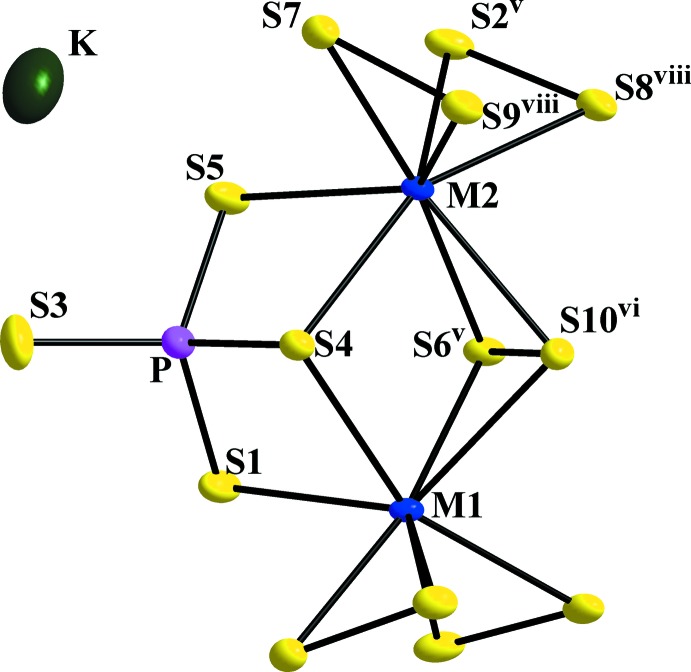
A view of the bicapped trigonal biprismatic [M2S12] unit (M=Nb/Ta) and its neighboring tetrahedral [PS4] groups. Yellow circles are S atoms, blue circles are Nb atoms, pink and dark green circles are P and K atoms, respectively. Displacement ellipsoids are drawn at the 50% probability level. [Symmetry code: (v) 0.5 - x, y, -0.5 + z; (vi) -0.5 + x, -y, z; (viii) 1 - x, -y, -0.5 + z]

**Fig. 2.**
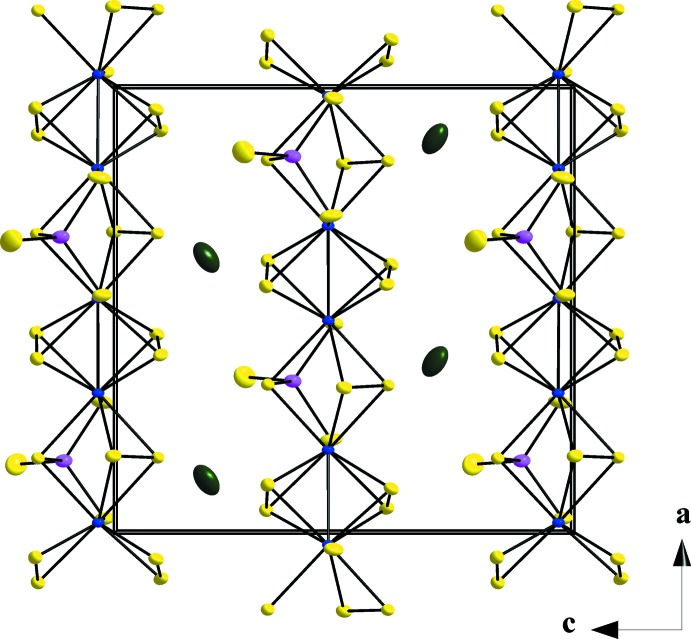
View of the KNb1.77Ta0.23PS10 down the b axis showing the one-dimensional nature of the compound. Atoms are as marked in Fig. 1.

## supplementary crystallographic information

### 1. Comment

A number of monovalent metal Nb thiophosphates have been investigated. Among them are NaNb_2_PS_10_ (Goh *et al.*, 2002), KNb_2_PS_10_ (Do & Yun, 1996), RbNb_2_PS_10_ (Kim & Yun, 2002), CsNb_2_PS_10_ (Kwak *et al.*, 2007), TlNb_2_PS_10_ (Bang *et al.*, 2008), Ag_0.88_Nb_2_PS_10_ (Do & Yun, 2009), K_0.34_Cu_0.5_Nb_2_PS_10_ (Kwak & Yun, 2008), K_0.5_Ag_0._Nb_2_PS_10_ (Dong *et al.*, 2005*a*), Rb_0.38_Ag_0.5_Nb_2_PS_10_ (Dong *et al.*, 2005*b*), Cs_0.5_Ag_0.5_Nb_2_PS_10_ (Park & Yun, 2010), and KNb_1.75_V_0.25_PS_10_ (Yu & Yun, 2011). As a result of efforts to find new phases in this family, we have found a mixed-metallic phase,. In this paper we report the synthesis and structure of another mixed-metallic quintenary thiophosphate, KNb_1.77_Ta_0.23_PS_10_.

The structure of KNb_1.77_Ta_0.23_PS_10_ is isostructural with KNb_2_PS_10_ and mixed-metallic KNb_1.75_V_0.25_PS_10_. Detailed description of the structure is given previously (Do & Yun, 1996; Yu & Yun, 2011). The title compound is made up of the usual bicapped trigonal biprismatic [*M*_2_S_12_] unit (*M*=Nb/Ta) and the tetrahedral [PS_4_] group. The *M* sites are occupied by the statistically disordered Nb(88.5%) and Ta(11.5%) atoms. The bicapped biprismatic [*M*_2_S_12_] units and its neighboring tetrahedral [PS_4_] groups are given in Figure 1. These [*M*_2_S_12_] units are linked together to form the one-dimensional chains, ∞^1^[*M*_2_PS_10_^-^] by sharing the S_2_^2-^ prism edge.

The *M* atoms associate in pairs with M—*M* interactions alternating in the sequence of one short (2.8939 (3) Å) and one long (3.7670 (3) Å) distances. The short distance is typical of Nb^4+^—Nb^4+^ bonding interactions (Angenault *et al.*, 2000). There are no interchain bonding interactions except the van der Waals forces and the K^+^ ions in this van der Waals gap stabilize the structure through the electrostatic interactions (Figure 2).

The structural studies of the three different crystals from the same reaction tube implied that the stoichiometry of each metal can vary, KNb_2 -_*_x_*Ta*_x_*PS_10_, 0.18≤*x*≤0.26 and they seem to form a random substitutional solid solution. However Ta analogue of this phase, ATa_2_PS_10_ has never been synthsized and thus the maximum *x* should be small. Finally, the classical charge balance of this phase can be represented by [K^+^][*M*^4+^]_2_[PS_4_^3-^][S_2_^2-^]_3_.

### 2. Experimental

The title compound, KNb_1.77_Ta_0.23_PS_10_ was prepared by the reaction of the elemental with the use of the reactive alkali metal halides-flux technique. A combination of the pure elements, Nb powder (CERAC 99.8%), Ta powder (CERAC 99.9%), P powder(Aldrich 99.9%), and S powder (Aldrich 99.999%) were mixed in a fused silica tube in a molar ratio of Nb: Ta: P: S = 1:1:1:10 with KCl (CERAC 99.9%). The mass ratio of the reactants and the alkali metal halides flux was 1:1. The tube was evacuated to 0.133 Pa, sealed and heated gradually (70 K/h) to 1073 K, where it was kept for 72 h. The tube was cooled to 473 K at 6 K/h and then was quenched to room temperature. The excess halides were removed with distilled water and black needle-shaped single crystals were obtained. The crystals are stable in air and water. Qualitative analysis of these crystals using XRF showed the presence of K, Nb, Ta, P, and S.

### 3. Refinement

The refinement of the model with occupational disorder on the *M* site caused significant decrease of the *R*-factor (wR2 = 0.042) in comparison if the full occupation by either metal had been considered (wR2 > 0.077). Also the displacement parameters in the disordered model became plausible. The disordered atoms were supposed to have the same displacement parameters. With the nonstoichiometric model, the parameter remained the same. The large anisotropic displacement parameters for alkali metals are also found in the related compounds such as KNb_2_PS_10_ (Do & Yun, 1996). The highest residual electron density is 0.40 Å from the M2 site and the deepest hole is 0.64 Å from the M1 site.

### Crystal data

**Table:**

KNb_1.77_Ta_0.23_PS_10_	*F*(000) = 1133
*M**_r_* = 596.52	*D*_x_ = 3.03 Mg m^−^^3^
Orthorhombic, *P**c**a*2_1_	Mo *K*α radiation, λ = 0.71073 Å
Hall symbol: P 2c -2ac	Cell parameters from 10926 reflections
*a* = 13.0049 (3) Å	θ = 3.1–27.4°
*b* = 7.5262 (2) Å	µ = 5.45 mm^−^^1^
*c* = 13.3616 (3) Å	*T* = 290 K
*V* = 1307.81 (6) Å^3^	Needle, black
*Z* = 4	0.32 × 0.06 × 0.04 mm

### Data collection

**Table:**

Rigaku R-AXIS RAPID S diffractometer	2974 independent reflections
Radiation source: sealed X-ray tube	2842 reflections with *I* > 2σ(*I*)
Graphite monochromator	*R*_int_ = 0.031
ω scans	θ_max_ = 27.4°, θ_min_ = 3.1°
Absorption correction: multi-scan (*ABSCOR*; Higashi, 1995)	*h* = −16→16
*T*_min_ = 0.724, *T*_max_ = 1.000	*k* = −9→9
12074 measured reflections	*l* = −17→17

### Refinement

**Table:**

Refinement on *F*^2^	1 restraint
Least-squares matrix: full	*w* = 1/[σ^2^(*F*_o_^2^) + (0.0132*P*)^2^ + 0.4965*P*] where *P* = (*F*_o_^2^ + 2*F*_c_^2^)/3
*R*[*F*^2^ > 2σ(*F*^2^)] = 0.019	(Δ/σ)_max_ = 0.001
*wR*(*F*^2^) = 0.040	Δρ_max_ = 0.40 e Å^−^^3^
*S* = 1.08	Δρ_min_ = −0.64 e Å^−^^3^
2974 reflections	Absolute structure: Flack (1983)
130 parameters	Absolute structure parameter: 0.102 (14)

### Special details

**Table:**

Geometry. All s.u.'s (except the s.u. in the dihedral angle between two l.s. planes) are estimated using the full covariance matrix. The cell s.u.'s are taken into account individually in the estimation of s.u.'s in distances, angles and torsion angles; correlations between s.u.'s in cell parameters are only used when they are defined by crystal symmetry. An approximate (isotropic) treatment of cell s.u.'s is used for estimating s.u.'s involving l.s. planes.
Refinement. Refinement of *F*^2^ against ALL reflections. The weighted *R*-factor *wR* and goodness of fit *S* are based on *F*^2^, conventional *R*-factors *R* are based on *F*, with *F* set to zero for negative *F*^2^. The threshold expression of *F*^2^ > 2σ(*F*^2^) is used only for calculating *R*-factors(gt) *etc*. and is not relevant to the choice of reflections for refinement. *R*-factors based on *F*^2^ are statistically about twice as large as those based on *F*, and *R*-factors based on ALL data will be even larger.

### Fractional atomic coordinates and isotropic or equivalent isotropic displacement parameters (Å^2^)

**Table:**

	*x*	*y*	*z*	*U*_iso_*/*U*_eq_	Occ. (<1)
K	0.38318 (10)	0.50405 (16)	0.30118 (10)	0.0643 (3)	
Nb1	0.024329 (14)	0.05323 (3)	0.03456 (2)	0.01373 (8)	0.8870 (16)
Ta1	0.024329 (14)	0.05323 (3)	0.03456 (2)	0.01373 (8)	0.1130 (16)
Nb2	0.313802 (14)	0.07133 (3)	0.03507 (2)	0.01349 (8)	0.8848 (16)
Ta2	0.313802 (14)	0.07133 (3)	0.03507 (2)	0.01349 (8)	0.1152 (16)
P	0.16059 (6)	0.40111 (13)	0.11222 (8)	0.02029 (19)	
S1	0.03152 (5)	0.39563 (11)	0.02331 (9)	0.0241 (2)	
S2	0.05537 (7)	0.15129 (15)	0.40771 (7)	0.0231 (2)	
S3	0.15181 (9)	0.58446 (14)	0.21735 (9)	0.0393 (3)	
S4	0.16755 (6)	0.14082 (12)	0.16603 (6)	0.01689 (19)	
S5	0.29233 (6)	0.41195 (10)	0.02861 (11)	0.0285 (2)	
S6	0.33066 (6)	0.05475 (12)	0.40561 (7)	0.0189 (2)	
S7	0.44837 (7)	0.13315 (14)	0.16957 (6)	0.0207 (2)	
S8	0.60107 (7)	0.10612 (14)	0.39842 (7)	0.0235 (2)	
S9	0.60965 (7)	0.11936 (13)	0.66622 (6)	0.0201 (2)	
S10	0.67408 (5)	0.16023 (11)	0.00053 (6)	0.01866 (18)	

### Atomic displacement parameters (Å^2^)

**Table:**

	*U*^11^	*U*^22^	*U*^33^	*U*^12^	*U*^13^	*U*^23^
K	0.0846 (8)	0.0454 (7)	0.0629 (8)	0.0038 (6)	−0.0317 (7)	0.0034 (6)
Nb1	0.00877 (11)	0.01781 (13)	0.01460 (12)	−0.00086 (7)	0.00002 (16)	0.00118 (14)
Ta1	0.00877 (11)	0.01781 (13)	0.01460 (12)	−0.00086 (7)	0.00002 (16)	0.00118 (14)
Nb2	0.00838 (11)	0.01692 (13)	0.01516 (12)	0.00106 (7)	0.00005 (15)	−0.00009 (14)
Ta2	0.00838 (11)	0.01692 (13)	0.01516 (12)	0.00106 (7)	0.00005 (15)	−0.00009 (14)
P	0.0158 (4)	0.0172 (4)	0.0279 (5)	0.0018 (3)	−0.0010 (4)	−0.0027 (4)
S1	0.0156 (3)	0.0208 (4)	0.0359 (6)	0.0022 (3)	−0.0026 (5)	0.0045 (5)
S2	0.0139 (4)	0.0342 (6)	0.0213 (5)	−0.0031 (4)	−0.0019 (4)	0.0071 (4)
S3	0.0450 (6)	0.0281 (5)	0.0449 (7)	0.0070 (5)	−0.0044 (5)	−0.0179 (5)
S4	0.0138 (4)	0.0200 (5)	0.0169 (4)	0.0004 (3)	0.0002 (3)	−0.0011 (4)
S5	0.0160 (3)	0.0192 (4)	0.0503 (6)	−0.0005 (3)	0.0068 (6)	0.0056 (6)
S6	0.0125 (4)	0.0275 (5)	0.0166 (4)	0.0000 (3)	0.0002 (3)	0.0027 (4)
S7	0.0168 (4)	0.0267 (5)	0.0185 (5)	0.0007 (4)	−0.0008 (4)	−0.0050 (4)
S8	0.0150 (4)	0.0354 (6)	0.0202 (5)	0.0047 (4)	0.0029 (4)	0.0084 (5)
S9	0.0166 (4)	0.0253 (5)	0.0183 (5)	0.0017 (4)	−0.0009 (4)	−0.0033 (4)
S10	0.0152 (4)	0.0169 (4)	0.0239 (4)	−0.0001 (3)	−0.0012 (3)	0.0018 (3)

### Geometric parameters (Å, º)

**Table:**

K—S3	3.2671 (16)	S2—S8^vi^	2.0302 (16)
K—S1^i^	3.2720 (16)	S2—Ta2^i^	2.4805 (9)
K—S9^ii^	3.3606 (15)	S2—Nb2^i^	2.4805 (9)
K—S7	3.4064 (16)	S2—Nb1^x^	2.5133 (10)
K—S2^iii^	3.7107 (16)	S2—Ta1^x^	2.5133 (10)
K—S6	3.7213 (15)	S2—K^xi^	3.7107 (16)
K—S3^iii^	3.7288 (19)	S3—K^xi^	3.7288 (19)
K—S10^iv^	3.7461 (16)	S6—S10^xii^	2.0568 (13)
Nb1—S8^v^	2.4753 (10)	S6—Ta1^i^	2.5545 (9)
Nb1—S7^vi^	2.4895 (9)	S6—Nb1^i^	2.5545 (9)
Nb1—S2^vii^	2.5133 (10)	S6—Ta2^i^	2.5568 (9)
Nb1—S9^v^	2.5255 (9)	S6—Nb2^i^	2.5568 (9)
Nb1—S6^v^	2.5545 (9)	S7—S9^viii^	2.0452 (15)
Nb1—S10^vi^	2.5653 (8)	S7—Ta1^ix^	2.4895 (9)
Nb1—S1	2.5831 (9)	S7—Nb1^ix^	2.4895 (9)
Nb1—S4	2.6438 (8)	S8—S2^ix^	2.0302 (17)
Nb1—Nb2^vi^	2.8939 (3)	S8—Nb1^i^	2.4753 (10)
Nb1—Ta2^vi^	2.8939 (3)	S8—Ta1^i^	2.4753 (10)
Nb2—S9^viii^	2.4742 (9)	S8—Nb2^xii^	2.5185 (10)
Nb2—S2^v^	2.4805 (9)	S8—Ta2^xii^	2.5185 (10)
Nb2—S8^viii^	2.5185 (10)	S9—S7^xii^	2.0452 (15)
Nb2—S7	2.5513 (9)	S9—Ta2^xii^	2.4742 (9)
Nb2—S6^v^	2.5568 (9)	S9—Nb2^xii^	2.4742 (9)
Nb2—S10^vi^	2.5597 (8)	S9—Nb1^i^	2.5255 (9)
Nb2—S5	2.5802 (8)	S9—Ta1^i^	2.5255 (9)
Nb2—S4	2.6369 (8)	S9—K^iv^	3.3606 (15)
Nb2—Ta1^ix^	2.8939 (3)	S10—S6^viii^	2.0568 (13)
Nb2—Nb1^ix^	2.8939 (3)	S10—Ta2^ix^	2.5597 (8)
P—S3	1.9725 (14)	S10—Nb2^ix^	2.5597 (8)
P—S5	2.0470 (14)	S10—Nb1^ix^	2.5653 (8)
P—S1	2.0568 (13)	S10—Ta1^ix^	2.5653 (8)
P—S4	2.0888 (14)	S10—K^ii^	3.7461 (16)
S1—K^v^	3.2720 (16)		
			
S3—K—S1^i^	132.02 (6)	S5—Nb2—Ta1^ix^	115.097 (18)
S3—K—S9^ii^	71.66 (3)	S4—Nb2—Ta1^ix^	138.44 (2)
S1^i^—K—S9^ii^	133.45 (4)	S9^viii^—Nb2—Nb1^ix^	55.46 (2)
S3—K—S7	101.77 (4)	S2^v^—Nb2—Nb1^ix^	55.12 (2)
S1^i^—K—S7	100.34 (4)	S8^viii^—Nb2—Nb1^ix^	53.89 (2)
S9^ii^—K—S7	114.03 (5)	S7—Nb2—Nb1^ix^	53.97 (2)
S3—K—S2^iii^	123.90 (4)	S6^v^—Nb2—Nb1^ix^	132.67 (2)
S1^i^—K—S2^iii^	67.78 (3)	S10^vi^—Nb2—Nb1^ix^	116.78 (2)
S9^ii^—K—S2^iii^	66.39 (3)	S5—Nb2—Nb1^ix^	115.097 (18)
S7—K—S2^iii^	128.37 (5)	S4—Nb2—Nb1^ix^	138.44 (2)
S3—K—S6	97.35 (4)	Ta1^ix^—Nb2—Nb1^ix^	0.000 (14)
S1^i^—K—S6	59.70 (3)	S3—P—S5	114.15 (6)
S9^ii^—K—S6	166.57 (4)	S3—P—S1	112.22 (6)
S7—K—S6	59.64 (3)	S5—P—S1	111.63 (7)
S2^iii^—K—S6	127.02 (4)	S3—P—S4	114.42 (7)
S3—K—S3^iii^	142.46 (6)	S5—P—S4	100.89 (5)
S1^i^—K—S3^iii^	84.85 (3)	S1—P—S4	102.44 (5)
S9^ii^—K—S3^iii^	87.91 (4)	P—S1—Nb1	90.91 (4)
S7—K—S3^iii^	57.68 (3)	P—S1—K^v^	104.02 (5)
S2^iii^—K—S3^iii^	71.02 (3)	Nb1—S1—K^v^	108.29 (4)
S6—K—S3^iii^	97.02 (4)	S8^vi^—S2—Ta2^i^	67.02 (4)
S3—K—S10^iv^	86.32 (4)	S8^vi^—S2—Nb2^i^	67.02 (4)
S1^i^—K—S10^iv^	65.83 (3)	Ta2^i^—S2—Nb2^i^	0.000 (15)
S9^ii^—K—S10^iv^	79.53 (3)	S8^vi^—S2—Nb1^x^	65.01 (4)
S7—K—S10^iv^	165.76 (4)	Ta2^i^—S2—Nb1^x^	70.83 (3)
S2^iii^—K—S10^iv^	51.37 (3)	Nb2^i^—S2—Nb1^x^	70.83 (3)
S6—K—S10^iv^	108.05 (4)	S8^vi^—S2—Ta1^x^	65.01 (4)
S3^iii^—K—S10^iv^	121.36 (4)	Ta2^i^—S2—Ta1^x^	70.83 (3)
S8^v^—Nb1—S7^vi^	111.20 (3)	Nb2^i^—S2—Ta1^x^	70.83 (3)
S8^v^—Nb1—S2^vii^	48.02 (4)	Nb1^x^—S2—Ta1^x^	0.000 (11)
S7^vi^—Nb1—S2^vii^	88.85 (3)	S8^vi^—S2—K^xi^	145.11 (5)
S8^v^—Nb1—S9^v^	91.47 (3)	Ta2^i^—S2—K^xi^	147.85 (4)
S7^vi^—Nb1—S9^v^	48.13 (3)	Nb2^i^—S2—K^xi^	147.85 (4)
S2^vii^—Nb1—S9^v^	107.82 (3)	Nb1^x^—S2—K^xi^	115.97 (4)
S8^v^—Nb1—S6^v^	89.43 (3)	Ta1^x^—S2—K^xi^	115.97 (4)
S7^vi^—Nb1—S6^v^	141.65 (3)	P—S3—K	93.51 (5)
S2^vii^—Nb1—S6^v^	81.51 (3)	P—S3—K^xi^	98.23 (5)
S9^v^—Nb1—S6^v^	168.03 (3)	K—S3—K^xi^	136.62 (6)
S8^v^—Nb1—S10^vi^	118.08 (3)	P—S4—Nb2	89.36 (4)
S7^vi^—Nb1—S10^vi^	94.41 (3)	P—S4—Nb1	88.54 (4)
S2^vii^—Nb1—S10^vi^	79.05 (3)	Nb2—S4—Nb1	91.02 (3)
S9^v^—Nb1—S10^vi^	140.41 (3)	P—S5—Nb2	91.88 (4)
S6^v^—Nb1—S10^vi^	47.37 (3)	S10^xii^—S6—Ta1^i^	66.59 (3)
S8^v^—Nb1—S1	79.67 (3)	S10^xii^—S6—Nb1^i^	66.59 (3)
S7^vi^—Nb1—S1	128.22 (3)	Ta1^i^—S6—Nb1^i^	0.000 (13)
S2^vii^—Nb1—S1	125.92 (3)	S10^xii^—S6—Ta2^i^	66.37 (3)
S9^v^—Nb1—S1	82.47 (3)	Ta1^i^—S6—Ta2^i^	94.95 (3)
S6^v^—Nb1—S1	85.96 (3)	Nb1^i^—S6—Ta2^i^	94.95 (3)
S10^vi^—Nb1—S1	125.95 (3)	S10^xii^—S6—Nb2^i^	66.37 (3)
S8^v^—Nb1—S4	155.83 (3)	Ta1^i^—S6—Nb2^i^	94.95 (3)
S7^vi^—Nb1—S4	86.48 (3)	Nb1^i^—S6—Nb2^i^	94.95 (3)
S2^vii^—Nb1—S4	153.05 (3)	Ta2^i^—S6—Nb2^i^	0.000 (6)
S9^v^—Nb1—S4	88.51 (3)	S10^xii^—S6—K	162.09 (5)
S6^v^—Nb1—S4	85.81 (3)	Ta1^i^—S6—K	96.98 (3)
S10^vi^—Nb1—S4	74.88 (3)	Nb1^i^—S6—K	96.98 (3)
S1—Nb1—S4	76.37 (3)	Ta2^i^—S6—K	110.13 (4)
S8^v^—Nb1—Nb2^vi^	55.28 (2)	Nb2^i^—S6—K	110.13 (4)
S7^vi^—Nb1—Nb2^vi^	55.97 (2)	S9^viii^—S7—Ta1^ix^	66.86 (4)
S2^vii^—Nb1—Nb2^vi^	54.06 (2)	S9^viii^—S7—Nb1^ix^	66.86 (4)
S9^v^—Nb1—Nb2^vi^	53.81 (2)	Ta1^ix^—S7—Nb1^ix^	0.000 (14)
S6^v^—Nb1—Nb2^vi^	134.55 (2)	S9^viii^—S7—Nb2	64.03 (3)
S10^vi^—Nb1—Nb2^vi^	121.05 (2)	Ta1^ix^—S7—Nb2	70.06 (2)
S1—Nb1—Nb2^vi^	110.949 (17)	Nb1^ix^—S7—Nb2	70.06 (2)
S4—Nb1—Nb2^vi^	138.22 (2)	S9^viii^—S7—K	132.92 (5)
S8^v^—Nb1—Ta2^vi^	55.28 (2)	Ta1^ix^—S7—K	159.16 (5)
S7^vi^—Nb1—Ta2^vi^	55.97 (2)	Nb1^ix^—S7—K	159.16 (5)
S2^vii^—Nb1—Ta2^vi^	54.06 (2)	Nb2—S7—K	110.02 (4)
S9^v^—Nb1—Ta2^vi^	53.81 (2)	S2^ix^—S8—Nb1^i^	66.97 (4)
S6^v^—Nb1—Ta2^vi^	134.55 (2)	S2^ix^—S8—Ta1^i^	66.97 (4)
S10^vi^—Nb1—Ta2^vi^	121.05 (2)	Nb1^i^—S8—Ta1^i^	0.000 (12)
S1—Nb1—Ta2^vi^	110.949 (17)	S2^ix^—S8—Nb2^xii^	65.06 (4)
S4—Nb1—Ta2^vi^	138.22 (2)	Nb1^i^—S8—Nb2^xii^	70.83 (3)
Nb2^vi^—Nb1—Ta2^vi^	0.000 (16)	Ta1^i^—S8—Nb2^xii^	70.83 (3)
S9^viii^—Nb2—S2^v^	110.53 (3)	S2^ix^—S8—Ta2^xii^	65.06 (4)
S9^viii^—Nb2—S8^viii^	91.66 (3)	Nb1^i^—S8—Ta2^xii^	70.83 (3)
S2^v^—Nb2—S8^viii^	47.92 (4)	Ta1^i^—S8—Ta2^xii^	70.83 (3)
S9^viii^—Nb2—S7	48.00 (3)	Nb2^xii^—S8—Ta2^xii^	0.000 (13)
S2^v^—Nb2—S7	88.20 (2)	S7^xii^—S9—Ta2^xii^	67.97 (4)
S8^viii^—Nb2—S7	107.81 (3)	S7^xii^—S9—Nb2^xii^	67.97 (4)
S9^viii^—Nb2—S6^v^	138.29 (3)	Ta2^xii^—S9—Nb2^xii^	0.000 (13)
S2^v^—Nb2—S6^v^	92.96 (3)	S7^xii^—S9—Nb1^i^	65.01 (3)
S8^viii^—Nb2—S6^v^	78.85 (3)	Ta2^xii^—S9—Nb1^i^	70.73 (2)
S7—Nb2—S6^v^	171.69 (3)	Nb2^xii^—S9—Nb1^i^	70.73 (2)
S9^viii^—Nb2—S10^vi^	91.05 (3)	S7^xii^—S9—Ta1^i^	65.01 (3)
S2^v^—Nb2—S10^vi^	121.90 (3)	Ta2^xii^—S9—Ta1^i^	70.73 (2)
S8^viii^—Nb2—S10^vi^	79.64 (3)	Nb2^xii^—S9—Ta1^i^	70.73 (2)
S7—Nb2—S10^vi^	137.57 (3)	Nb1^i^—S9—Ta1^i^	0.000 (11)
S6^v^—Nb2—S10^vi^	47.41 (3)	S7^xii^—S9—K^iv^	141.46 (5)
S9^viii^—Nb2—S5	130.06 (4)	Ta2^xii^—S9—K^iv^	149.10 (4)
S2^v^—Nb2—S5	79.06 (4)	Nb2^xii^—S9—K^iv^	149.10 (4)
S8^viii^—Nb2—S5	123.38 (4)	Nb1^i^—S9—K^iv^	124.00 (4)
S7—Nb2—S5	85.22 (4)	Ta1^i^—S9—K^iv^	124.00 (4)
S6^v^—Nb2—S5	86.92 (3)	S6^viii^—S10—Ta2^ix^	66.22 (3)
S10^vi^—Nb2—S5	126.41 (3)	S6^viii^—S10—Nb2^ix^	66.22 (3)
S9^viii^—Nb2—S4	86.29 (3)	Ta2^ix^—S10—Nb2^ix^	0.000 (17)
S2^v^—Nb2—S4	154.41 (3)	S6^viii^—S10—Nb1^ix^	66.04 (3)
S8^viii^—Nb2—S4	154.60 (3)	Ta2^ix^—S10—Nb1^ix^	94.62 (3)
S7—Nb2—S4	89.49 (3)	Nb2^ix^—S10—Nb1^ix^	94.62 (3)
S6^v^—Nb2—S4	85.91 (3)	S6^viii^—S10—Ta1^ix^	66.04 (3)
S10^vi^—Nb2—S4	75.09 (3)	Ta2^ix^—S10—Ta1^ix^	94.62 (3)
S5—Nb2—S4	75.35 (3)	Nb2^ix^—S10—Ta1^ix^	94.62 (3)
S9^viii^—Nb2—Ta1^ix^	55.46 (2)	Nb1^ix^—S10—Ta1^ix^	0.000 (16)
S2^v^—Nb2—Ta1^ix^	55.12 (2)	S6^viii^—S10—K^ii^	94.94 (4)
S8^viii^—Nb2—Ta1^ix^	53.89 (2)	Ta2^ix^—S10—K^ii^	136.76 (3)
S7—Nb2—Ta1^ix^	53.97 (2)	Nb2^ix^—S10—K^ii^	136.76 (3)
S6^v^—Nb2—Ta1^ix^	132.67 (2)	Nb1^ix^—S10—K^ii^	113.42 (3)
S10^vi^—Nb2—Ta1^ix^	116.78 (2)	Ta1^ix^—S10—K^ii^	113.42 (3)

Symmetry codes: (i) −*x*+1/2, *y*, *z*+1/2; (ii) −*x*+1, −*y*+1, *z*−1/2; (iii) *x*+1/2, −*y*+1, *z*; (iv) −*x*+1, −*y*+1, *z*+1/2; (v) −*x*+1/2, *y*, *z*−1/2; (vi) *x*−1/2, −*y*, *z*; (vii) −*x*, −*y*, *z*−1/2; (viii) −*x*+1, −*y*, *z*−1/2; (ix) *x*+1/2, −*y*, *z*; (x) −*x*, −*y*, *z*+1/2; (xi) *x*−1/2, −*y*+1, *z*; (xii) −*x*+1, −*y*, *z*+1/2.
